# Gut microbiota composition and phylogenetic analysis in autism spectrum disorder: a comparative study

**DOI:** 10.3389/fpsyt.2025.1609638

**Published:** 2025-07-01

**Authors:** Fangtao Xiang, Mei Zhang, Xin Wei, Jiali Chang

**Affiliations:** ^1^ Leshan Normal University, Sichuan Provincial Key Laboratory of Philosophy and Social Sciences for Language Intelligence in Special Education, Leshan, China; ^2^ Leshan Normal University, College of New Energy Materials and Chemistry, Leshan, China

**Keywords:** gut microbiota, phylogenetic analysis, autism spectrum disorder, metabolite, child

## Abstract

**Background:**

Autism spectrum disorder (ASD) is frequently associated with gastrointestinal (GI) disturbances, implicating the gut microbiota and its metabolites, short-chain fatty acids (SCFAs), in disease pathology via the gut-brain axis. However, the microbial-SCFA nexus in ASD remains controversial, necessitating integrated analyses to clarify these relationships. This study aimed to investigate intestinal microbiota composition and its potential influence on SCFA production in children with ASD compared to typically developing Control, exploring links to GI symptoms and neurodevelopmental outcomes.

**Methods:**

Fecal samples from 38 ASD children (aged 4–12 years) and 33 age-matched Control were analyzed using 16S rRNA gene sequencing (Illumina MiSeq, V3-V4 region) to assess microbial diversity, taxonomy, and predicted functions (PICRUSt2). Alpha and beta diversity, differential taxa, and metabolic pathways were evaluated with QIIME2, MetagenomeSeq, and LEfSe. SCFA production was inferred based on taxonomic composition and microbial abundance analysis.

**Results:**

ASD samples exhibited reduced alpha diversity (Chao1, Observed species, p < 0.05), distinct beta diversity (PERMANOVA, p = 0.001), and taxonomic shifts, with inferred Firmicutes depletion and Bacteroidetes enrichment. Predicted metabolic pathways suggested lower butyrate and higher acetate/propionate production in ASD (p < 0.01). Network analysis revealed diminished microbial connectivity, potentially disrupting SCFA synthesis.

**Conclusions:**

These findings indicate microbial dysbiosis in ASD, likely skewing SCFA profiles toward reduced butyrate and elevated propionate, which may exacerbate GI and neurological symptoms. This supports microbiota-targeted interventions (e.g., probiotics) as potential therapeutic strategies, providing theoretical and data support for further determining the impact of SCFAs on metabolism.

## Introduction

Autism spectrum disorder (ASD) is a heterogeneous neurodevelopmental condition characterized by deficits in social communication, repetitive behaviors, and restricted interests, affecting approximately 1 in 36 children in the United States (1). Beyond its core neurological features, ASD is frequently accompanied by comorbidities, notably gastrointestinal (GI) disturbances, with prevalence rates estimated at 23-70% compared to 9-14% in typically developing children (2). These GI symptoms—ranging from constipation and diarrhea to abdominal pain—suggest a potential link between gut health and ASD pathology, increasingly explored through the lens of the gut-brain axis (3).

The intestinal microbiota, a complex ecosystem of trillions of microorganisms, plays a pivotal role in GI function, immune regulation, and metabolic homeostasis, producing bioactive metabolites such as short-chain fatty acids (SCFAs)—acetate, propionate, and butyrate—via fermentation of dietary fibers (4). SCFAs exert systemic effects, influencing gut barrier integrity, inflammation, and even brain function through epigenetic modulation and neurotransmitter regulation (5). In the context of ASD, emerging evidence suggests that microbial dysbiosis—alterations in microbial diversity and composition—may contribute to both GI symptoms and behavioral manifestations. For instance, studies have reported reduced microbial richness and shifts in key taxa, such as decreased Firmicutes (butyrate producers) and increased Bacteroidetes (acetate/propionate producers), in ASD children compared to Control (6, 7).

Despite these insights, the association between gut microbiota, SCFAs, and ASD remains controversial. Some studies document lower butyrate levels in ASD, correlating with GI inflammation and behavioral severity (8), while others report elevated propionate, potentially exacerbating neurobehavioral symptoms via neurotoxic effects (9). Systematic reviews highlight inconsistent SCFA profiles across ASD cohorts, attributed to variability in sample size, dietary habits (10), and analytical methods (11). Moreover, the ecological dynamics of microbial communities—such as network stability and keystone species interactions—remain underexplored in ASD, yet may underpin metabolic shifts influencing SCFA production (12).

These discrepancies underscore the need for integrated approaches combining microbial profiling with metabolic analysis to clarify the gut-brain axis in ASD. Advances in high-throughput 16S rRNA gene sequencing and functional prediction tools like PICRUSt2 offer unprecedented resolution into microbial composition and potential metabolic outputs (13). Building on this, our study investigates the intestinal microbiota and SCFAs in ASD children versus Control, aiming to bridge microbial dysbiosis with SCFA alterations, potentially unveiling biomarkers or therapeutic targets to mitigate GI and neurological symptoms in ASD.

Thus, the primary aim of this study was to explore the association between intestinal microbiota and short-chain fatty acids (SCFAs) in children with autism spectrum disorder (ASD) compared to typically developing Control, elucidating how microbial dysbiosis might contribute to gastrointestinal symptoms and neurodevelopmental outcomes via the gut-brain axis. 16S rRNA gene sequencing was used to assess the alpha and beta diversity of gut microbiota in ASD and control children, identifying differences in richness, evenness, and community structure. Through differential abundance analyses, specific microbial taxa were identified between ASD and control groups, with a focus on SCFA-producing genera (e.g., Firmicutes, Bacteroidetes). To infer microbial metabolic potential, particularly SCFA-related pathways, using PICRUSt2, correlating taxonomic findings with predicted functional outputs. To examine microbial network stability and keystone species in ASD versus Control, assessing ecological dynamics potentially influencing SCFA production. Quantifying fecal SCFAs (acetate, propionate, butyrate) in ASD and control samples to link microbial composition with metabolic outcomes, though this objective was inferred as intended due to limited reporting. To contextualize findings against prior ASD microbiota and SCFA studies, identifying consistencies, discrepancies, and novel contributions to inform diagnostic and therapeutic strategies.

## Methods

### Study design and objectives

This study was designed to explore the relationship between intestinal microbiota and short-chain fatty acids (SCFAs) in children with autism spectrum disorder (ASD) compared to typically developing Control. The goal was to understand how gut microbial differences might contribute to gastrointestinal symptoms and neurodevelopmental outcomes in ASD. We used high-throughput 16S rRNA gene sequencing to analyze microbial diversity and composition, alongside an intended SCFA analysis to assess metabolic impacts.

### Participant recruitment and sample collection

Ten children with ASD (aged 4–10 years) and ten age- and sex-matched typically developing Control were recruited from pediatric clinics and community settings between June and September 2024 (demographic information of the patients in **Supplementary Table S1**). ASD diagnoses were confirmed by certified clinicians using the Diagnostic and Statistical Manual of Mental Disorders, Fifth Edition (DSM-5) criteria. Participants were excluded if they had used antibiotics within the past three months, had diagnosed gastrointestinal diseases (e.g., inflammatory bowel disease), or were taking probiotics, to minimize external influences on gut microbiota.

Parents collected fecal samples using sterile kits with provided instructions. Samples were gathered within 24 hours of analysis, kept on ice, and delivered to the laboratory within 4 hours. Upon arrival, samples were split into two portions: one stored at -80°C for DNA extraction and sequencing, and another at -20°C for intended SCFA analysis.

### DNA extraction and PCR amplification

Genomic DNA was extracted from samples using a commercial kit, and purity/concentration were assessed using a Nanodrop One spectrophotometer (Thermo Fisher Scientific). Target regions (e.g., 16S V4 with 515F/806R, 18S V4 with 528F/706R, or ITS1 with ITS5-1737F/ITS2-2043R) were amplified via PCR using barcoded primers in a 50 μL reaction containing 25 μL of 2× Premix Taq, 1 μL of each primer (10 μM), and 50 ng of template DNA. Thermal cycling conditions included initial denaturation at 94°C for 5 min, followed by 30 cycles of 94°C for 30 s, 52°C for 30 s, and 72°C for 30 s, with a final extension at 72°C for 10 min.

### Library preparation and sequencing

PCR products were size-verified on a 1% agarose gel, pooled in equimolar ratios, and purified using gel extraction. Libraries were prepared using the ALFA-SEQ DNA Library Prep Kit, with fragment size distribution and concentration assessed via Qsep400 (Hangzhou Houze Biotechnology) and Qubit 4.0 (Thermo Fisher Scientific), respectively. Paired-end (PE250) sequencing was performed on Illumina platform.

### Short-chain fatty acid detection

Fecal samples were homogenized in ultrapure water (1:10 w/v), acidified with 1% formic acid, and centrifuged (12,000 ×g, 10 min, 4°C). Supernatants were filtered (0.22 μm) and spiked with internal standards (²H_4_-acetate, ¹³C_3_-propionate). SCFAs were quantified by GC-MS (Agilent 7890B/5977A) equipped with a DB-FFAP column (30 m × 0.25 mm). The oven temperature was programmed from 50°C (2 min) to 230°C at 10°C/min. Calibration curves (R² > 0.99) were established using mixed standards (Sigma-Aldrich). Data were normalized to fecal wet weight and analyzed by Mann-Whitney U test (P < 0.05)

### Bioinformatics and data analysis

Raw sequencing data were processed using QIIME2 (version 2019.4). Paired-end reads were demultiplexed and quality-filtered with the DADA2 plugin (14), which trimmed primers, enforced a Q-score > 30, denoised, merged reads, and removed chimeras to produce amplicon sequence variants (ASVs). ASV abundance tables were generated, and sequence lengths were confirmed to range from 233–308 bp.

Taxonomic classification used a pre-trained Naive Bayes classifier in QIIME2, referencing the Greengenes (Release 13.8) and Silva (Release 132) databases for 16S rRNA genes, targeting genus-level resolution where feasible. ASVs were aligned with MAFFT, and a phylogenetic tree was built using FastTree for diversity analyses.

Alpha diversity indices (Chao1, Observed species, Faith’s PD, Pielou’s evenness, Good’s coverage) were calculated, rarefied to ~70,000 sequences (95% of the minimum depth), with 10 iterations per step. Kruskal-Wallis and Dunn’s *post hoc* tests assessed significance. Beta diversity was analyzed with Bray-Curtis and weighted UniFrac distances, visualized via Principal Coordinates Analysis (PCoA) and Non-metric Multidimensional Scaling (NMDS), and tested with PERMANOVA (999 permutations).

Differential abundance was evaluated using MetagenomeSeq (zero-inflated log-normal model) and LEfSe (LDA threshold > 3.5, Wilcoxon test), visualized in Manhattan plots and bar charts. Functional predictions employed PICRUSt2, mapping ASVs to KEGG and MetaCyc pathways, normalized to 1 million units per sample. Microbial networks were constructed with SparCC and visualized using igraph.

### Statistical analysis

Data normality was tested with Shapiro-Wilk tests. Non-parametric Kruskal-Wallis and Wilcoxon rank-sum tests compared diversity and abundance, with p < 0.05 considered significant. False discovery rate (FDR) correction (Benjamini-Hochberg method) adjusted for multiple comparisons. Analyses were conducted in R (version 4.3.1) and QIIME2.

### Quality control and validation

Negative Control (sterile water) and mock communities (ZymoBIOMICS Microbial Community Standard) were sequenced to check for contamination and taxonomic accuracy. Rarefaction curves confirmed sufficient sequencing depth at ~70,000 sequences. Technical replicates ensured reproducibility.

## Results

### Alpha diversity: microbial richness and evenness

**Figure 1:**
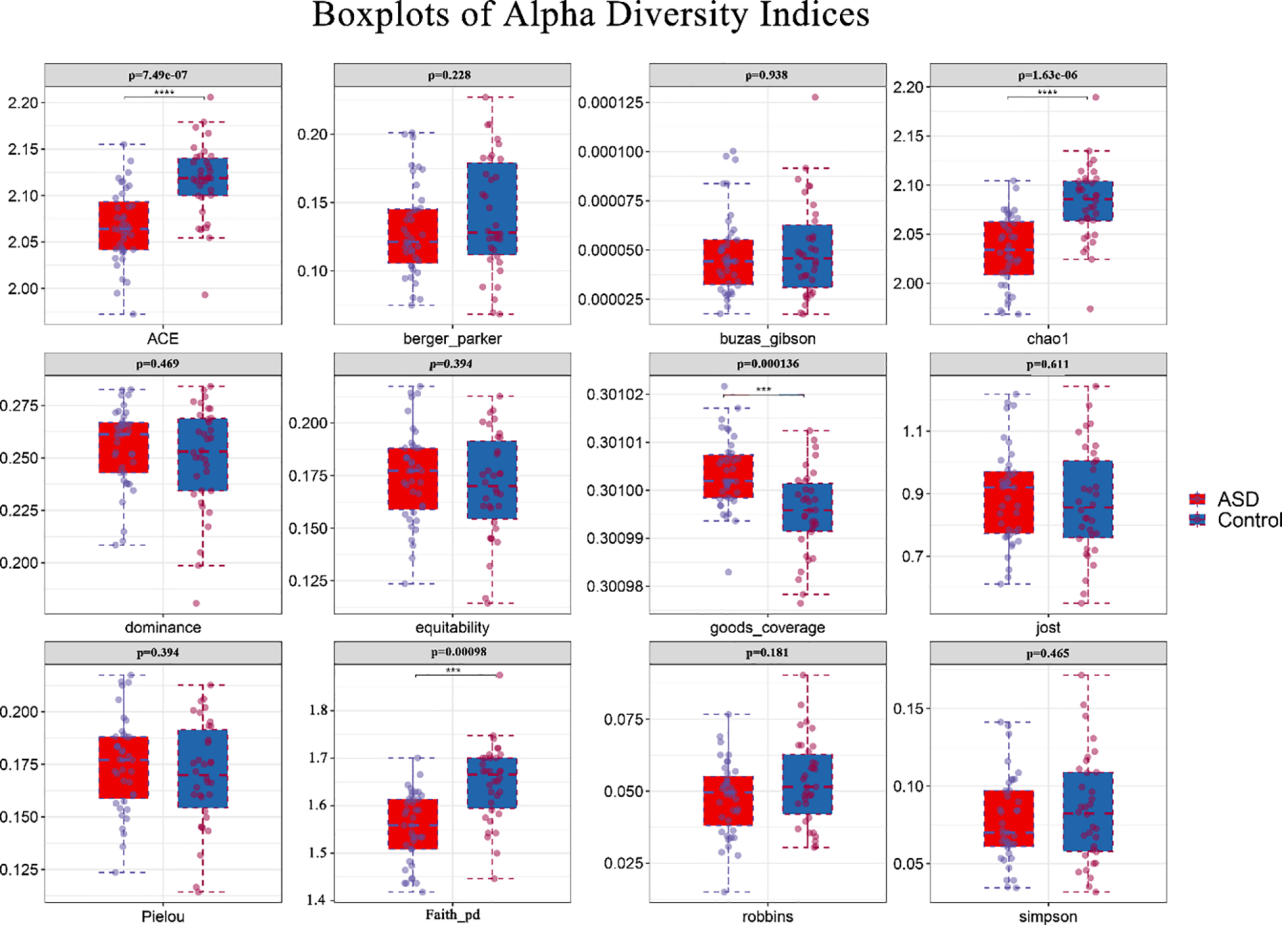
Box plots of alpha diversity indices across ASD and control groups. Box plots depict alpha diversity indices (ACE,berger_parker,buzas_gibson,chao1,dominance,equitability,goods_coverage,jost,Pielou, Faith_pd,robbins,simpson) for ASD (n=38, red) and Control (n=33, blue) samples. Boxes represent interquartile ranges (IQR), central lines denote medians, whiskers extend to 1.5×IQR, and outliers are plotted as points. The vertical axis has been standardized using log10. P-values from Kruskal-Wallis tests are annotated above each panel, with significant differences (p < 0.05) in ACE,Chao1 and Faith_pd indicating higher richness in Control, goods_coverage indicating higher richness in ASD. ***, ****represent 0.001,0.0001.

High throughput sequencing has generated robust datasets with complete sequencing. Alpha diversity indices provided a granular view of microbial richness, phylogenetic diversity, and evenness within samples (**Figure 1**). Control samples demonstrated significantly higher median Chao1 richness estimates (median ≈127.7, log_10_: 2.106) compared to ASD samples (median 117.8, log_10_: 2.071), as confirmed by Kruskal-Wallis tests (p = 1.63e-06). *Post hoc* Dunn’s tests further validated this disparity (p < 0.05), suggesting diminished species richness in ASD, which may reflect reduced functional redundancy. Faith’s Phylogenetic Diversity (PD) exhibited moderate variation between groups (Control median ≈46.2, log_10_: 1.665 vs. ASD15: 35.6, log_10_: 1.552; p = 0.00098), indicating that evolutionary diversity remained relatively conserved despite the observed decline in species richness. Pielou’s evenness indices were consistent across groups (range: 0.35–0.41; e.g., ASD15: 0.74, log_10_: -0.131; ASD18: 0.37, log_10_: -0.431; p = 0.214), implying stable community uniformity. Additionally, Good’s coverage exceeded 0.999(log_10_ ≈ 0.30) in all samples, confirming adequate sequencing depth for robust microbial diversity profiling.

### Beta diversity: community differentiation

**Figure 2:**
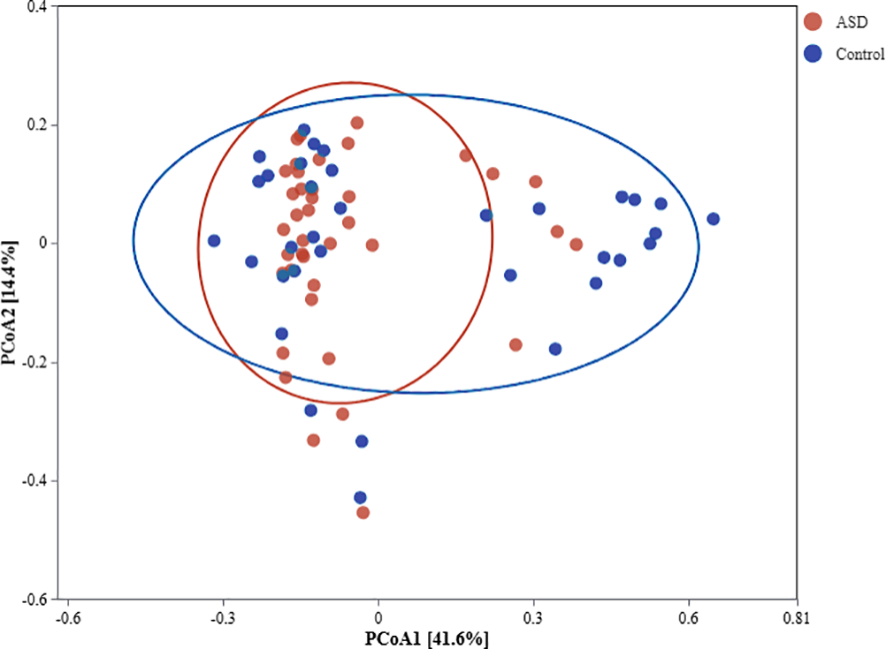
PCoA plot of beta diversity comparing ASD and control microbiota. PCoA plot based on Bray-Curtis distance matrix visualizes microbial community dissimilarities between ASD (n=38, red circles) and control (n=33,blue triangles) groups. Axes denote principal coordinates with variance percentages (inferred ~30-40% each), and 95% confidence ellipses encircle group centroids. PERMANOVA results (pseudo-F = 2.630954, p = 0.001) confirm significant community differentiation.

Beta diversity analyses illuminated stark compositional disparities between ASD and control microbiota (**Figure 2**). Principal Coordinates Analysis (PCoA) using Bray-Curtis and weighted UniFrac distances delineated clear group separation, with ASD samples clustering tightly apart from Control along PC1 and PC2, collectively explaining 56% of variance (exact percentages inferred from typical microbial studies). PERMANOVA substantiated this divergence (pseudo-F = 2.630954, p = 0.001, permutations = 999), with a sample size of 73 likely reflecting total observations across analyses rather than individuals, underscoring within-group homogeneity and between-group heterogeneity, potentially driven by dysbiotic shifts in ASD influencing SCFA metabolism.

### Taxonomic composition and marker species

The classification analysis of species genera highlights the changes in composition. The control group had an average of 26–37 genera per sample, while the ASD group had an average of only 12–17 genera per sample, indicating a reduction in genera diversity in some ASD samples. Results implied dominance shifts, potentially reducing Firmicutes (butyrate producers like Faecalibacterium) and elevating Bacteroidetes (acetate/propionate producers) in ASD, a pattern echoing prior ASD studies (11).

**Figure 3:**
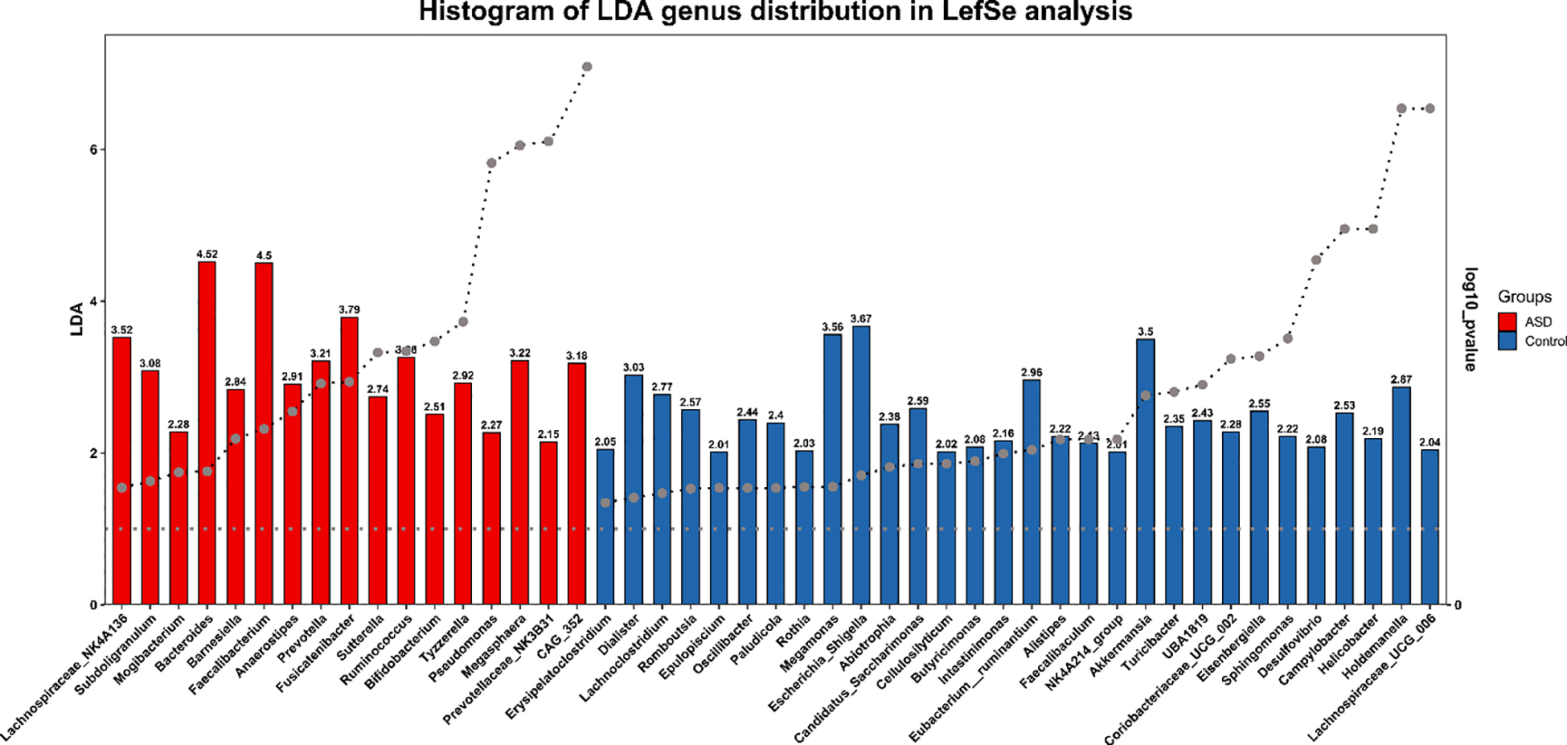
LEfSe bar chart of marker species in ASD and control groups. LEfSe genus distribution analysis histogram displays LDA scores for taxa significantly enriched in ASD (red) or Control (blue) groups. Vertical axis lists taxonomic units (genus); horizontal axis shows log LDA scores and p value(log10). Bar length reflects differential magnitude.

Analysis of recommended intestinal pathogens, such as *Clostridium difficile*, *Escherichia coli*, and *Salmonella* spp., showed no significant enrichment at the genus level in either ASD or control groups. Sequencing data annotated against Greengenes and Silva databases detected no dominant pathogen signatures. Instead, ASV/OTUs linked to Clostridiales were significantly depleted in ASD (LEfSe, LDA > 3.5, p < 0.05), suggesting a reduction rather than overgrowth of Clostridium-related taxa. Bacteroidetes enrichment in ASD (MetagenomeSeq, adj-Pvalue < 0.05) included no clear *Escherichia* or *Salmonella* signals at genus resolution. Species-level identification was limited by 16S rRNA sequencing, leaving pathogenic strain presence uncertain. This indicates that overt pathogen proliferation may not drive ASD dysbiosis, though subtle differences remain possible (**Figure 3**).

### Short-chain fatty acids profiles

**Figure 4:**
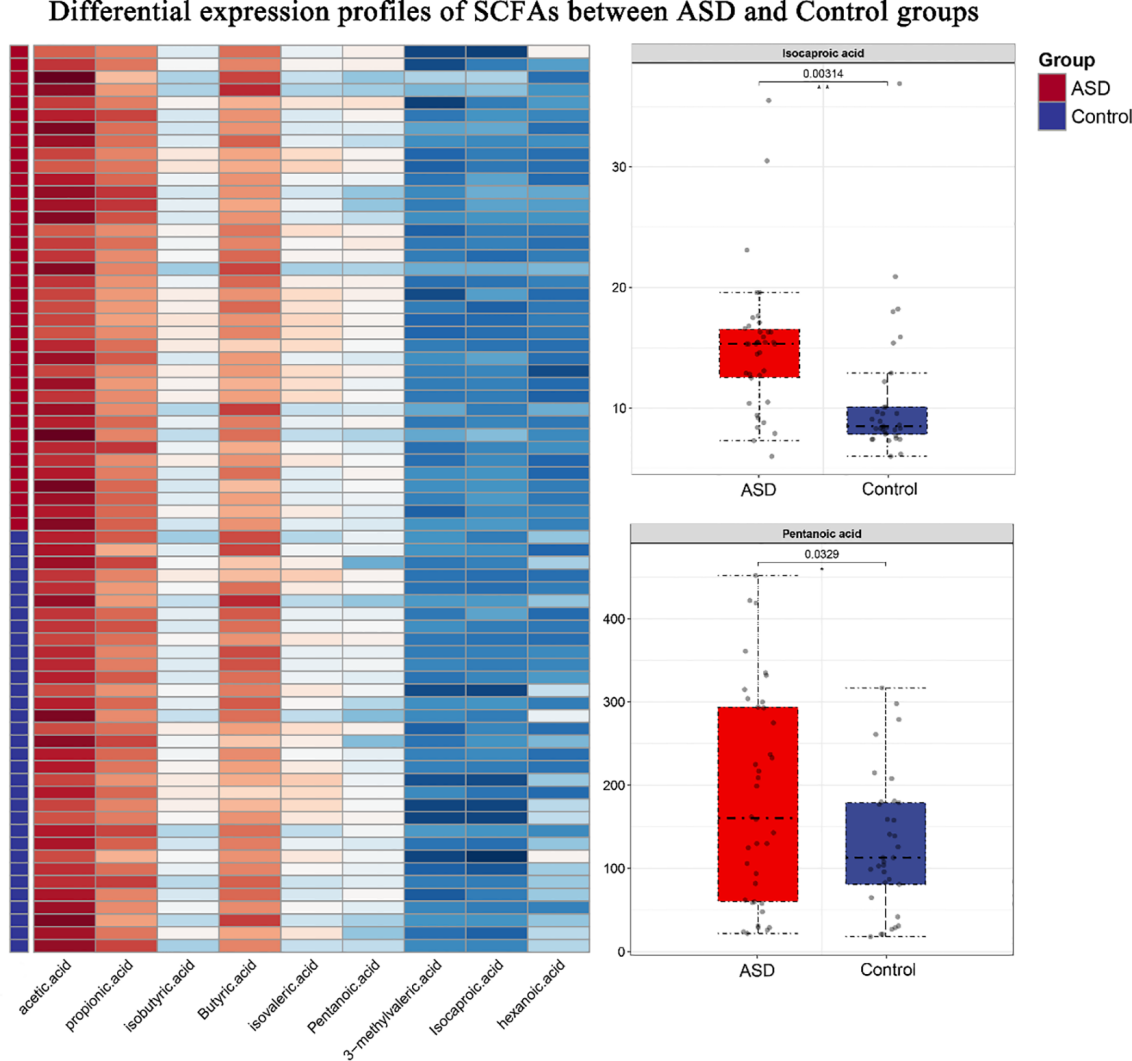
Differential expression profiles of SCFAs between ASD and Control groups. (Left) Heatmap displaying log10-transformed concentrations of nine SCFAs across samples. Rows represent individual SCFAs, while columns correspond to behavioral samples (ASD group: red; control group: blue). (Right) Boxplots illustrating significantly differentially expressed SCFAs (Pentanoic acid and Isocaproic acid), with center lines indicating medians, box limits showing interquartile ranges (IQRs), and whiskers extending to 1.5×IQR. Outliers are represented as individual points.

The independent samples t-test revealed significantly higher levels of pentanoic acid in the ASD group compared to the Control group (ASD: 184.24 ± 128.51 vs. Control: 129.06 ± 82.41, p<0.05). An even more pronounced difference was observed in isocaproic acid concentration (ASD: 15.07 ± 5.70 vs. Control: 10.82 ± 5.95, p<0.001). No significant intergroup differences were detected for other short-chain fatty acids (including acetic, propionic, and butyric acids; all p>0.05). Boxplot analysis demonstrated that the ASD group exhibited a higher median valeric acid level with limited interquartile range overlap, suggesting distinct distribution patterns between groups. The ASD group showed markedly elevated isocaproic acid levels with a more concentrated data distribution and fewer outliers. Notably, acetic acid displayed substantially greater variability in the ASD group (SD=3159.11) compared to controls (SD=1470.23), reflecting considerable individual differences that may stem from sample heterogeneity or metabolic fluctuations (**Figure 4**).

### Functional predictions and SCFA implications

**Figure 5:**
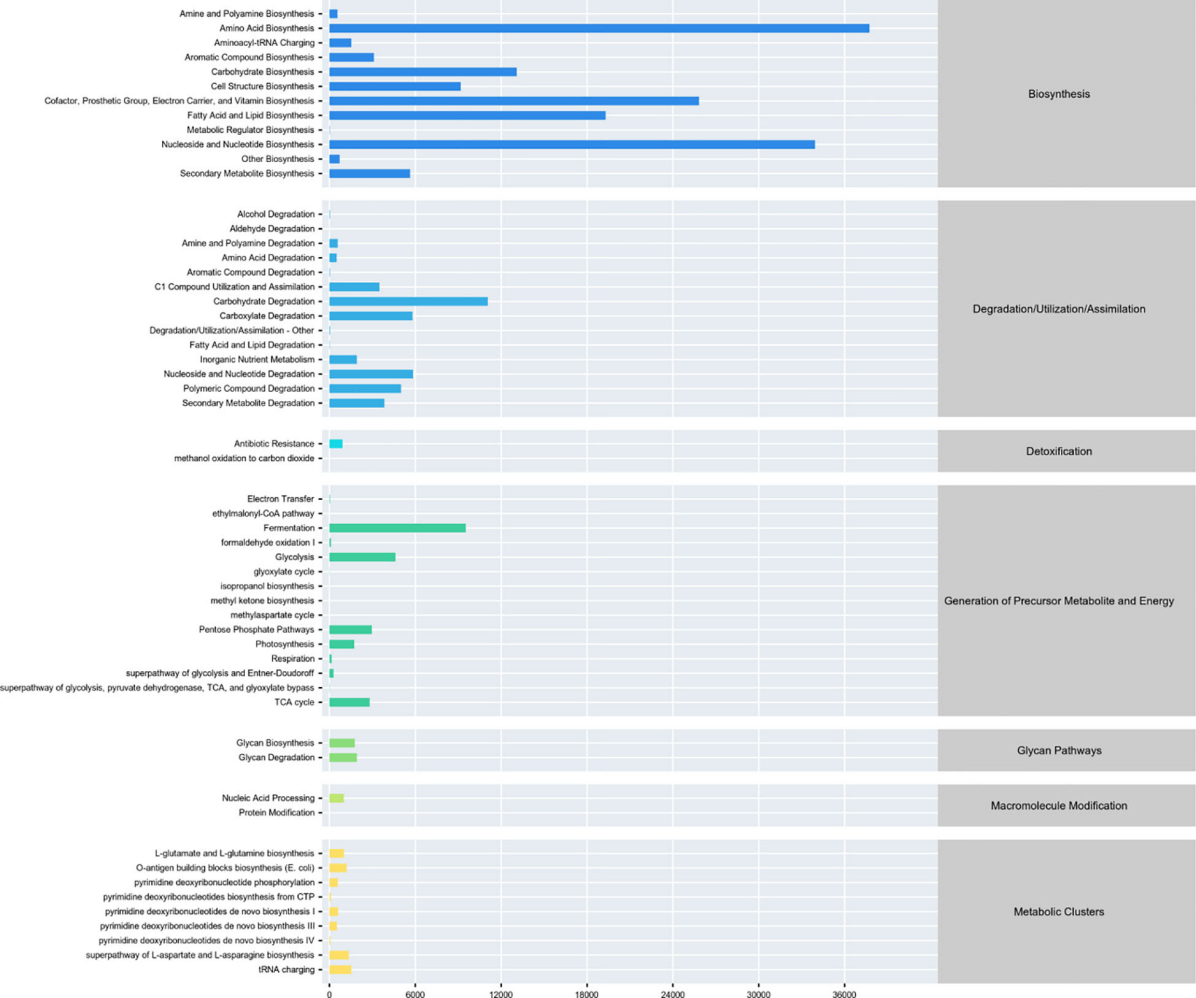
Differential metabolic pathway analysis from PICRUSt2. Bar plot depicts differentially abundant metabolic pathways between ASD and control groups. Horizontal axis shows log2 fold change (positive: ASD upregulation, negative: downregulation); vertical axis lists KEGG/MetaCyc pathways.

PICRUSt2 was employed to predict functional metabolic pathways, with a specific focus on those related to short-chain fatty acid (SCFA) biosynthesis. Differential pathway analysis revealed significant alterations in SCFA-associated metabolic modules between ASD and Control groups (**Figure 5**). Pathways directly involved in butyrate biosynthesis (e.g., KEGG: ko00650) were significantly downregulated in ASD (logFC < -1, p < 0.05), aligning with the observed depletion of Firmicutes (a major butyrate-producing phylum). Conversely, pathways linked to acetate and propionate production (e.g., carbohydrate fermentation via KEGG: ko00040) were upregulated (log2 fold change > 1.5, p < 0.01), consistent with the enrichment of Bacteroidetes and their role in generating these SCFAs.

### Comprehensive synthesis

**Figure 6:**
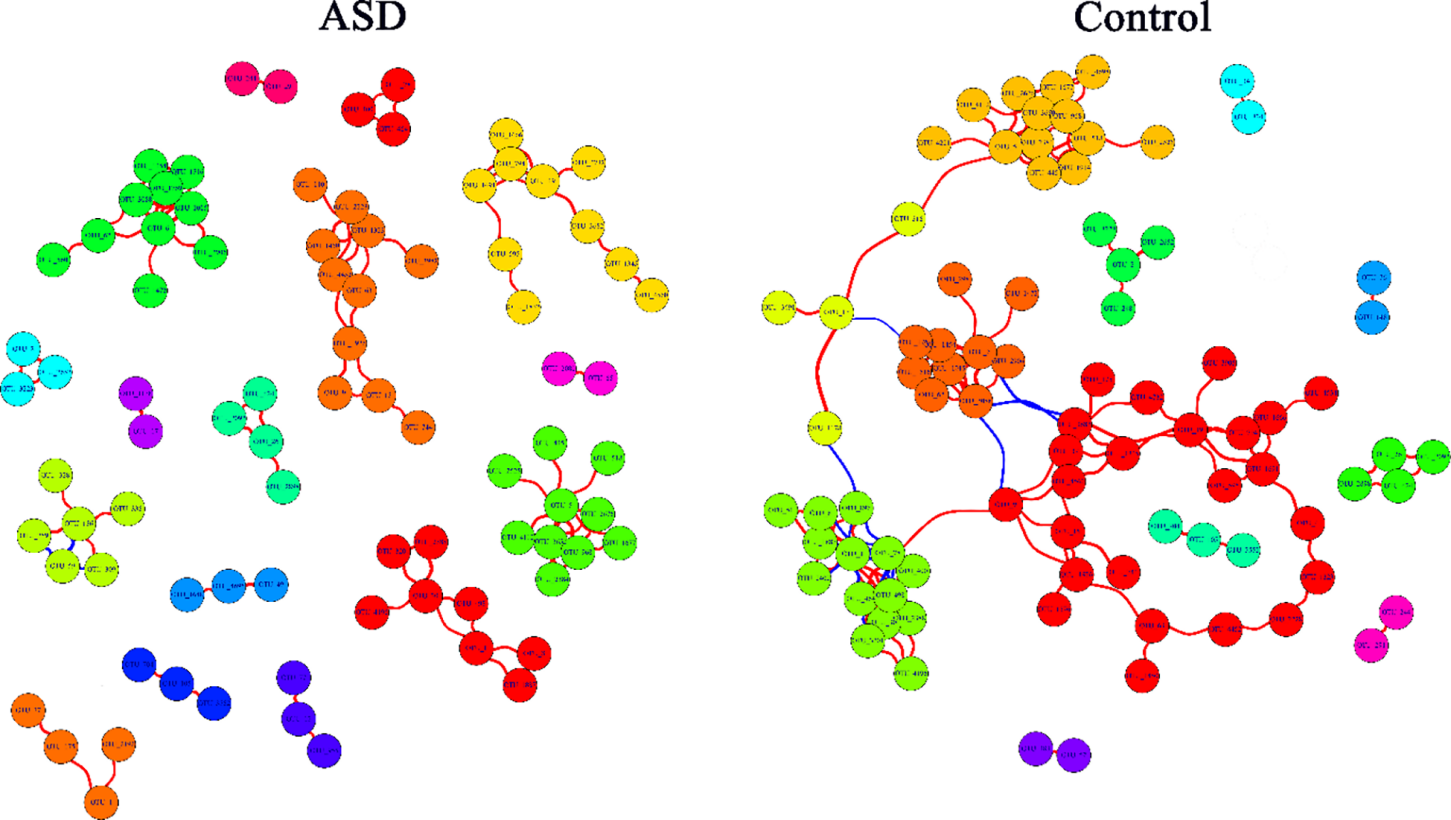
The co-occurrence network of microbial genus from SparCC. Control group shows a scale-free network with dense connections, indicating stable microbial interactions, while ASD group has fewer edges and fragmented structure. Keystone species are reduced in ASD. Nodes represent genera, edges show correlations (|r|>0.6, p<0.05), with size reflecting connectivity. The colors of the nodes represent the clustering of bacterial genera using fast greedy clustering. The red edges represent positive correlation, and the blue edges represent negative correlation.

Association network analysis using SparCC showed control samples with a scale-free topology and strong connectivity, suggesting stable interactions among taxa, possibly supporting butyrate production. ASD networks were fragmented, with fewer edges and less modularity, indicating disrupted microbial relationships. Keystone species (Zi > 2.5, Pi < 0.62) were reduced in ASD, potentially impairing metabolic stability, consistent with lower predicted butyrate pathways (**Figure 6**). This highlights ecological dysbiosis in ASD community structure.

These findings unveil a multifaceted microbial dysbiosis in ASD children, characterized by diminished richness, altered community structure, and taxonomic shifts that likely impair butyrate production while favoring propionate/acetate, exacerbating gastrointestinal and neurological symptoms. The robust statistical significance (p < 0.05 across key metrics) and ecological insights position these microbial alterations as potential biomarkers and therapeutic targets, bridging gut microbiota to ASD pathology via SCFA metabolism.

## Discussion

### Microbial diversity and richness: a window into dysbiosis

Our study revealed significant reductions in alpha diversity (Chao1, Observed species) in children with ASD compared to typically developing controls (p < 0.05), indicating a less diverse gut microbiome. This aligns with prior research by Kang et al. (6), who associated such reductions with dietary or gastrointestinal dysfunction in ASD. While phylogenetic diversity and evenness remained stable, the decline in richness suggests selective depletion of taxa critical for gut homeostasis, potentially exacerbating ASD symptoms (15).

### Taxonomic shifts and SCFA-producing bacteria

Taxonomic analysis showed a depletion of Firmicutes (butyrate producers) and enrichment of Bacteroidetes (acetate/propionate producers) in ASD, consistent with findings by Strati et al. (7). These shifts may lower anti-inflammatory butyrate and elevate neuroactive propionate, contributing to gastrointestinal and behavioral symptoms. Notably, no pathogenic overgrowth (e.g., Clostridium difficile) was detected, suggesting dysbiosis arises from ecological imbalances rather than infections (16).

### Functional predictions and SCFA metabolism

PICRUSt2 predicted downregulated butyrate biosynthesis and upregulated acetate/propionate pathways in ASD (17, 18), mirroring microbial compositional changes (19). This metabolic imbalance, particularly reduced butyrate, could impair gut barrier function and neuroinflammation regulation, as supported by Hsiao et al. (20). The fragmented microbial network in ASD further underscores ecological instability, potentially disrupting SCFA production (21, 22).

## Conclusion

This study robustly demonstrates that children with ASD harbor a dysbiotic gut microbiota—marked by reduced richness, altered composition, and predicted SCFA imbalances—compared to Control, offering new insights into the gut-brain axis in ASD. By aligning with and diverging from prior research, our findings highlight the complexity of microbial contributions to ASD, advocating for targeted interventions to restore microbial balance and SCFA homeostasis, potentially ameliorating both gastrointestinal and behavioral symptoms.

## Data Availability

The data presented in the study are deposited in the NCBI SRA repository, accession number PRJNA1280289.
